# Evaluating the reliability of gestalt quality ratings of medical education podcasts: A METRIQ study

**DOI:** 10.1007/s40037-020-00589-x

**Published:** 2020-06-03

**Authors:** Jason M. Woods, Teresa M. Chan, Damian Roland, Jeff Riddell, Andrew Tagg, Brent Thoma

**Affiliations:** 1 Section of Emergency Medicine, Children’s Hospital Colorado, University of Colorado School of Medicine, Aurora, Colorado USA; 2 Division of Emergency Medicine, Department of Medicine, McMaster University, Hamilton, ON Canada; 3 Paediatric Emergency Medicine, Leicester Royal Infirmary, University of Leicester, Leicester, UK; 4 Department of Clinical Emergency Medicine, Keck School of Medicine, University of Southern California, Los Angeles, USA; 5 University of Melbourne, Melbourne, Australia; 6 Footscray Hospital, Melbourne, Australia; 7 Department of Emergency Medicine, University of Saskatchewan, Saskatoon, Saskatchewan Canada

**Keywords:** Podcast, Gestalt, Reliability, FOAMEd

## Abstract

**Introduction:**

Podcasts are increasingly being used for medical education. Studies have found that the assessment of the quality of online resources can be challenging. We sought to determine the reliability of gestalt quality assessment of education podcasts in emergency medicine.

**Methods:**

An international, interprofessional sample of raters was recruited through social media, direct contact, and the extended personal network of the study team. Each participant listened to eight podcasts (selected to include a variety of accents, number of speakers, and topics) and rated the quality of that podcast on a seven-point Likert scale. Phi coefficients were calculated within each group and overall. Decision studies were conducted using a phi of 0.8.

**Results:**

A total of 240 collaborators completed all eight surveys and were included in the analysis. Attendings, medical students, and physician assistants had the lowest individual-level variance and thus the lowest number of required raters to reliably evaluate quality (phi >0.80). Overall, 20 raters were required to reliably evaluate the quality of emergency medicine podcasts.

**Discussion:**

Gestalt ratings of quality from approximately 20 health professionals are required to reliably assess the quality of a podcast. This finding should inform future work focused on developing and validating tools to support the evaluation of quality in these resources.

**Electronic supplementary material:**

The online version of this article (10.1007/s40037-020-00589-x) contains supplementary material, which is available to authorized users.

## Introduction

Open educational resources such as blogs and podcasts are increasingly prevalent in emergency medicine [1]. A drastic increase in their availability [1] and use [2] has coincided with the rise of concerns regarding their quality [3, 4]. Podcasts are commonly utilized by emergency medicine residents in the United States [5], Canada [2], the United Kingdom, and Australia [6] and have been shown to affect clinical decision making in some settings [5]. Despite their potential impact on patient care, we are unaware of any studies which formally investigate their quality.

Studies have found the assessment of the quality of online resources to be difficult [7–11]. Resources have been developed to assist trainees and clinicians to assess the quality of blog posts [7, 12–14] but podcast listeners have had to rely upon their own gestalt to evaluate the quality of these resources. As the reliability of gestalt is limited by each individual’s unique experience and learning needs [15, 16], the effectiveness of this approach is unclear.

We hypothesized that, like the gestalt evaluation of blog post quality [8, 12], clinicians will have broadly discrepant perspectives on the quality of individual podcasts. To test this hypothesis, we recruited an international, multidisciplinary sample of emergency clinicians to rate the quality of podcasts. If we are correct, our findings would provide empirical evidence to support concerns regarding users’ ability to distinguish between high- and low-quality podcasts and suggest the need to develop of podcast-specific evaluation tools.

## Methods

This study was deemed exempt from ethical review (Research Ethics Board, University of Saskatchewan, BEH 17-170). This work was carried out in accordance with the Declaration of Helsinki (http://www.wma.net/en/30publications/10policies/b3), including, but not limited to, there being no potential harm to participants, the anonymity of participants was guaranteed with regards to the results, and informed consent of participants was obtained.

### Participant recruitment and retention

We recruited participants using the METRIQ study method [17] as described in greater detail elsewhere [18]. As the goal of this study was to obtain a representative sample of the virtual community of practice that concerns itself with medical education podcasts, we intentionally utilized an open process for participant recruitment. Communities of practice are made up of people who “share a common interest in a topic, and who deepen their knowledge and expertise by interacting on an ongoing basis” [19]. Dubé et al. further delineated the term *virtual communities of practice* to indicate the same shared features but where the primary interaction is in a virtual environment [20]. Our international authorship team promoted study participation by reaching out to their personal networks via email and their online community of practice via Facebook, Twitter, and WhatsApp. We also sent collaborators from the METRIQ blog study a recruitment email. Investigators from Canada, the United States, the United Kingdom, Australia, and South Africa conducted the study which aimed to recruit an international study population. We did not specify any particular level of expertise in either podcast listening or evaluation of medical education materials. We did this intentionally to recruit a sample representative of the general medical education podcasts listenership, rather than to recruit a cohort of content experts.

We directed potential participants to https://METRIQstudy.org where they completed an intake form. Potential participants received a link to an initial survey within 24 h of completing the intake form. After it was completed [18], participants were directed to a series of eight podcasts and asked to respond to a brief survey after listening to each. We sent participants up to four reminders to complete each survey that were spaced out by 1–2 weeks. Participants who completed all surveys were included in the analyses and recognized as contributors to the METRIQ podcast study.

### Survey design and podcast selection

The eight podcasts we selected for this study were sampled from websites tracked by the Social Medial Index [21, 22]. We chose this number of podcasts because we felt that this was the most that would be feasible for volunteer participants to complete as part of the study. The podcasts were intentionally selected to include a variety of accents (two each recorded by native speakers from Canada, the United States, the United Kingdom, and Australia) and number of speakers (four had a single speaker and four had multiple speakers). All podcasts were approximately 20 min in length (range 17 to 23 min, mean 21.6 min). To reduce the likelihood that participants had already listened to the podcast, we preferentially selected recently published podcasts. We organized the podcasts on a single podcast channel that could be accessed online or added to whatever podcast application was regularly used by the participants. This allowed participants to access the podcasts included in the study in the listeners’ usual fashion. We received consent for the use of their content from the owner of each of the podcasts.

After listening to each podcast, participants responded to the question: Please indicate the extent to which you agree or disagree with the following statement: “This podcast episode was of high quality for medical education” with responses on a Likert scale from 1 (strongly disagree) to 7 (strongly agree). This question was modified from other studies evaluating the gestalt quality of open educational resources [13, 14, 23, 24] to be specific to podcasts.

### Data analysis

We exported raw survey data from FluidSurveys and calculated descriptive statistics using Microsoft Excel. Calculations were conducted on both the full rater population and within all subgroups consisting of more than two raters. Generalizability studies (G-studies), analysis of variance (ANOVA), and decision studies (D-studies) were conducted using G‑String IV (Hamilton, ON, Canada). The D‑studies determined the number of raters needed to achieve a phi of ≥0.80 [25].

## Results

A total of 240 collaborators were included in the analysis. The study population included physicians and physician-trainees (residents/medical students), nurses, prehospital providers, and physician assistants. As only a single emergency medicine pharmacist participated so their data were excluded from the analysis. Tab. 1 depicts the subgroups of raters.

**Table 1:** Summary data for podcast raters

Subgroups	*n*	Age, mean (SD)	Gender
All participants	240	33.1 (7.9)	56.0% male
			43.6% female
			0.4% prefer not to disclose
Attending or consultant physicians	73	38.0 (7.0)	69.9% male
			30.1% female
Pre-hospital care providers	27	31.9 (7.0)	70.4% male
			29.6% female
Medical students	67	26.6 (3.8)	49.3% male
			50.7% female
Nurse & nurse practitioners	22	39.3 (9.6)	27.3% male
			68.2% female
			4.5% prefer not to disclose
Physician assistants	11	39.1 (7.7)	72.7% male
			27.3% female
Residents/Registrars/Fellows	40	30.8 (3.4)	42.5% male
			57.5% female
Location	240	Canada 122 (50.6%)	
		United States 59 (24.5%)	
		Europe 32 (13.3%)	
		Oceana 14 (5.8%)	
		Africa 9 (3.7%)	
		South America 4 (1.7%)	
		Asia 1 (0.4%)	

There was variation in the average quality ratings for the podcasts with the lowest rated 4.5 and the highest 6.2 on the 7‑point Likert scale. The ANOVA, generalizability study, and decision study are shown in Tab. 2. The ANOVA found that prehospital providers and the nursing group had the greatest individual-level variance. As the G‑study phi co-efficient computes a measure of reliability of all raters in each group, it is affected by the number of raters (more raters, higher phi), the D‑study calculation (how many raters needed from that group for a phi ≥0.80) is a better comparison between the groups. The D‑study found that physician assistants (13), medical students (15), and attendings (18) required the lowest numbers of raters to achieve adequate reliability while nurse/nurse practitioners had the highest (33).

**Table 2:** Variance, generalizability, and decision studies

		ANOVA study			Generalizability study	Decision study
	Number of raters in group	% Variance due to podcast	% Variance due to rater in group	% Variance due to Podcast by rater (p × r) interaction	G‑coefficient (Phi) for the whole group	How many raters in this group needed to have a Phi coefficient ≥0.80
All raters	240	17.0%	9.9%	73.2%	0.98	20
Attendings	73	17.8%	8.6%	73.7%	0.94	18
Residents, registrars, & fellows	27	15.0%	9.4%	75.6%	0.88	23
Medical students	67	21.6%	8.7%	69.7%	0.95	15
Nurses & nurse practitioners	22	9.9%	31.8%	58.3%	0.77	27
Physician assistants	11	23.3%	16.4%	60.3%	0.81	13
Prehospital care providers	40	10.8%	12.8%	76.3%	0.77	33

## Discussion

This study evaluated the overall and subgroup-specific reliability of gestalt ratings of medical education podcast quality. Our results suggest that, with enough raters, gestalt can be used to determine the quality of educational podcasts. However, the ratings of small numbers of raters are insufficiently reliable. Further, our findings emphasize the need to develop tools that support podcast quality evaluation, which could build on the advances in quality evaluation of other open educational resources [12–14, 24].

Some subgroups were more reliable than others. The difference in magnitude of the D‑studies for each group may relate to different interpretations of quality within each group that could stem from higher heterogeneity in these populations (e.g. resident/registrar/fellow, nurse/nurse practitioner, and prehospital provider populations, may have more varied training experience than the other groups). Intuitively this makes sense, since a first-year postgraduate trainee (PGY1) will be unlikely to have the same perspective as a PGY5 or Fellow, who are much closer to the culmination of their training; whereas third- and fourth-year medical students may have very similar educational needs. Similarly, the nurse (consisting of both nurses and nurse practitioners) and prehospital (consisting of primary and advanced or critical care paramedics) clinician populations would arguably have greater diversity in training background than the physician assistant population which achieved the highest level of reliability. Other studies have only been conducted in physicians and physician trainees but have not consistently replicated this finding. Krishnan et al. [11] found that trainees were less reliable than attendings when rating blog posts while Thoma et al. [7] did not find a substantial difference.

Our findings are substantively different from those evaluating other open educational resources such as blog posts. A previous D‑study found that raters evaluating blog post quality using gestalt require at least 43 raters to achieve adequate reliability [7]. All of the subgroups in our study performed better than this when evaluating podcasts. While we can only speculate regarding why this was the case, it may be that podcasts are experienced more consistently than blog posts. It is also notable that this previous study was conducted in a more homogenous population (only medical students, emergency medicine residents, and emergency medicine attendings) so the opposite result (less reliability in this population) was more likely based upon the group composition alone.

The major strength of our study is its inclusion of a large and diverse sample of participants from multiple health professions that increases its generalizability. Further, this is the first study investigating the quality of online educational resources which included the perspectives of non-physician health practitioners. Our results demonstrate the variability in which clinicians evaluate podcasts and support the need for the development of evaluation tools that would guide the clinicians using them.

### Limitations

As a survey-based study that utilized a social media recruitment strategy, this work has several limitations. The population that we targeted for recruitment were existing medical podcast listeners, so it is unlikely that these results would be generalizable to non-listeners and may be less relevant to podcast listeners who are not active on social media. As nearly 10% of the participants owned, operated, edited, or managed their own podcasts, our participants likely have more experience with podcasts than a general population of podcast listeners. The selected podcasts were delivered only in English and the participants were primarily from English-speaking countries, so the findings cannot be extended to other languages. Lastly, our pragmatic study design did not allow us to ensure that our participants listened to each podcast episode in full. While this behavior mirrors the real-world behavior of clinicians who listen primarily while exercising and commuting, it may affect their ability to reliably assess quality [26].

## Conclusions

Gestalt ratings of quality from approximately 20 health professionals are required to reliably assess the quality of a podcast. This finding should inform future work focused on developing and validating tools to support the evaluation of these resources.

## Caption Electronic Supplementary Material

The METRIQ Study Collaborators
