# Supplementary material for: Evaluating the reliability of gestalt quality ratings of medical education podcasts: A METRIQ study
Source: Perspect Med Educ. 2020 Jun 3;9(5):302–6. doi: 10.1007/s40037-020-00589-x (PMC7550476; doi:10.1007/s40037-020-00589-x)
Supplement: Supplementary file 1 — The METRIQ Study Collaborators [file 40037_2020_589_MOESM1_ESM.docx]

**Evaluating the Reliability of Gestalt Quality Ratings of Medical Education Podcasts: A METRIQ Study**

The METRIQ Study Collaborators

The METRIQ Podcast Study Collaborators (in alphabetical order by last name):

Peter Agaba, Mohammed M Aldawood, Mohammed Makki Aldawood, Mathew Alex, Salma Ali, Laura Allan, Mohammed Almuhanna, Ashwini Amaratunga, Jeremy Amayo, Natalie Anderson, Omar Anjum, Mads Astvad, Arden Azim, Patrick Bafuma, Michelle Bailey, Steve Baker, Kimberly Baldino, Joanna Banaszek, Alex Barber, David Barton, Alan Batt, Rachel Baumgartner, Jared Baylis, Taylor Bechamp, Stéphanie Beckett, Chris Bell, Antônio Felippe Benini, Temesgen Beyene, Meghan Bhatia, Richard Biedermann, Märta Björling, Jessica Blackbourn, Michael Blanchard, Iria Miguens Blanco, Brandon Blondeau, Bryan Boling, Andrew Bowman, Emma Bradshaw, Victoria Brazil, Ian Breden, Page Bridges, Joshua Britton, Robert Bruce, Stevan Bruijns, Ineke Brummer, Wisarut Bunchit, Kevin Burns, Mike Butler, Alan C. Taylor, Avery Callahan, Federico Carini, Christina Castillo, Ryan Chadwick, Christie Wing Long Chan, Kathryn Chan, Teresa Chan, Alvin Chin, Rachel Christian, Alex Christiansen, Alyssa Cocchiara, Isabelle Colmers-Gray, Joe Colucci, Roberto Cosentini, Stefan Cowtan, Avery Crocker, Henry Cullen, Andrew D'alessandro, Bagonza I Kenneth Daniel, Aaron Danielson, Christi Denton, Alixe Dick, Susana Garcia Diez, Tatjana Dill, Matt Dionne, Anthony Doherty, Danielle Donoghue, Maia Dorsett, Doug Lynch, Hugo Dowd, Philippe Dubuc-Gaudreau, Taylor Duda, Justin Dueweke, William Dunkerley, Kevin Durr, Marcia Edmonds, Sylvia Egels, Kirsty Ellis, Elaine Erasmus, Rebecca Erker, Caley Flynn, Mark Frederikse, Ushira Ganas, Joe Ghorayeb, Scott Goerzen, Siew Pei Goh, Mark Goichman, Amanda Goodfellow, Puja Gopal, Michael Gottlieb, Paul Grinzi, Andrew Grock, Tanner Gronowski, Catriona Gunn, Adrianna Gunton, Andrew Hall, Benjamin Hardy, Lukas Hashem, Erfun Hatam, Philip Hehn, John Heisler, Chloe Henley, Suelin Hilbert, Sakura Hingley, Amy Ho, Nicholas Hochfelden, Brenden Hoff, Caroline Hoogerheide, Timothy Horeczko, Dirk Houtman, Lamont Hunter, Qasim Hussain, Elizabeth Namugaya Igaga, Pholaphat Charles Inboriboon, Aaron Inouye, Irene Ostapowich, Palbha Jain, Jesse Jamieson, Pieter Jan, Suneth Jayasekara, Dayna Jaynstein, Maria Jirwe, Levi Johnston, Will Johnston, Kalanzi Joseph, Clint Kalan, Kelly Kaley, Drew Kalnow, Annika Kamberelis, Ramya Kancherla, Aravind Kashyap, Gerben Keijzers, Catherine Kerr, Jaasmit Khurana, Jerry Kim, Katie Knight, Lisa Knijnenberg, Justin Koh, Melodie Kolmetz, Daniel Korpal, James Kozak, Alexander Krois, Ivanna Kruhlak, Natalia Krupin, Michael I Kruse, Stephen Kydd-Hindelang, Simon Lainh, Chip Lange, Bryan Laviolette, Jacqui Le Geyt, Andrew Leach, Jennifer Leckie, Charles Lei, Edgar Lei, Vivian Lei, Haakon Lenes, Nilantha Lenora, Mason Leschyna, Tim Leung, Ian Lewins, Resa E Lewiss, Winny Li, Kelly Lien, Brodie Lipon, Andrew Little, Jonathan Little, Steve Liu, Megan Loucks, Stephanie Louka, Jessica Gy Luc, S. Luckett-Gatopoulos, Kristopher Maday, Sonali Mantoo, Paige Mason, Rebecca Maxwell, Cian Mcdermott, Michael Mcdonnell, Jonathan Mcghee, Sean Mcintosh, Susan Mclellan, Amy Mcnaughtan, Carolyn Mcquarrie, Katie Mcpadden, Therese Mead, Patrick Meloy, Maartje Melse, Donna Mills, Brendan Moore, Justin Morgenstern, Lee Morissette, Sarah Mott, Pinaki Mukherji, Lisa Murphy, Victoria Myers, Vanessa Naidoo, Gabriel Najarro, Meera Nariadhara, Annet Alenyo Ngabirano, Dan Nguyen, Taylor Nikel, Anton Nikouline, Mais Nuaaman, Sean Nugent, Dayle Ostapiuk, Nadine Ouderkirk, Rob Paquin, Alim Pardhan, Ravi Parhar, Quinten Paterson, Christine Patterson, Caroline Chandler Pedrozo, Gina Pellerito, Juliana Pereira, Brock Phillips, Sarah Plante, Zoe Polsky, Dawn Prall, Michael Prats, Joel Privé, Gregor Prosen, Henrique Alencastro Puls, Vishal Puri, Chichen Qiu, Tanis Quaife, Md, Salim R. Rezaie, Dillan Radomske, John Reagan, Elissa Remmer, Jeff Riddell, Milan Ridderikhof, Jamie Riggs, Brendan Riordan, Doreen Rivera, Isabel Rizor, Genevieve Robinson, Damian Roland, Kaye Rolls, Stuart Rose, Keith Rosenberg, Kyle Roshan Wong, Paul Ross, Brenda Ruberto, Evan Russell, Mackenzie Russell, Syed S Ahmed, Abdul Sattar Abdul Safras, Eleni Salakidou, Erin Sandilands, Michelle L Santos, Alan Sazama, Owen Scheirer, Rebecca Schulman, Paul Schunk, Makamu Sebakeng, Ashley Selvig, Cornelius Sendagire, Alex Senger, Parisa Shahrabadi, Julia Sheffield, David Sheps, Jack Shier, Seyara Shwetz, Mansoor Siddiqui, Teresa Siefke, Sarah Simons, Adam Singer, Gabilan Sivapatham, Shyam Sivasankar, Sarah Skolfield, Sam Smith, Paula Sneath, Cassandra Sobkiw-Kurtz, Robert Soegtrop, Peter Speare, Jan Spicer, Anna Spinaze, Adrienne Stedford, James Stempien, Britni Sternard, Brittany Stewart, Katie Stuart, Danielle Swart, Colleen Sweeney, Henry Swoboda, Andrew Tagg, Christopher Tait, Nikhil Tambe, Elisha Targonsky, Michael Taylor, Robert Taylor, Luis Eduardo Vargas Téllez, Brent Thoma, Brent Thoma, Megan Thoma, Adam Thomas, Gareth Thomas, Liam Thorley, Gerhard Tiwald, Sydney Todorovich, Meriel Tolhurst-Cleaver, Luz-Patricia Torres, Max Tory, Charlene A Traynor, Seth Trueger, Troy Turner, Amol Utrankar, Italo Vasquez Vargas, Christopher Velasquez, René Verbeek, Femke Verbree, Jacqueline Vulto, Sonja Wakeling, Ateshia Walker, Gregory Wanner, Kevin Webb, Brittni Webster, Kristen Weersink, Hao Wei Chen, Meghan Wentzell, James Werbicki, Scott J Wieters, Gabby Wilcox, Penny Wilson, Nelson Wong, Mark Woodcroft, Jason Woods, Rob Woods, Nichole Woodward, Fred Wu, Craig Wylie, Kevin Wyne, Lisa Tai-Ling Ying, Louise Hammer Yndigegn, Heinri Zaayman, Leah Zhao, Katina Zheng, Alex Zozula
